# Crystal structure of *catena*-poly[bis­(*N*,*O*-di­methyl­hydroxyl­ammonium) [di-μ-bromido-di­bromido­stannate(II)]]

**DOI:** 10.1107/S2056989024012027

**Published:** 2025-01-01

**Authors:** Valerii Y. Sirenko, Mircea-Odin Apostu, Irina A. Golenya, Dina D. Naumova, Sofiia V. Partsevska

**Affiliations:** a Department of Chemistry, Taras Shevchenko National University of Kyiv, Volodymyrska str. 64/13, 01601 Kyiv, Ukraine; b Department of Chemistry, Faculty of Chemistry, Al. I. Cuza University of Iasi, Carol I Blvd. 11, Iasi 700506, Romania; Vienna University of Technology, Austria

**Keywords:** metal halides, stannate, *N*,*O*-di­methyl­hydroxyl­amine, tin(II) bromide, Hirshfeld surface analysis, crystal structure

## Abstract

The title compound, {(C_2_H_8_NO)_2_[SnBr_4_]}_*n*_, is a layered hybrid perovskite crystallizing in the monoclinic space group *C*2/*c*. The asymmetric unit consists of one H_3_C—O—NH_2_^+^—CH_3_ cation (Me_2_HA^+^), one Sn^II^ atom located on a twofold rotation axis, and two Br atoms. The Sn^II^ atom has a distorted octa­hedral coordination environment formed by the bromido ligands. The {SnBr_6_} units corner-share their equatorial Br atoms, forming infinite mono-layers that extend parallel to the *ab* plane. These inorganic layers are sandwiched by the organic Me_2_HA^+^ cations organized in double-layers; stacking of the layers is along the *c-*axis direction. Consecutive inorganic layers, separated by the organic cations, are shifted relative to each other along the *b*-axis direction. Specifically, the Sn^II^ atom in one inorganic layer is offset by 3.148 Å along the *b* axis relative to the Sn^II^ atom in an adjacent inorganic layer. The *N*,*O*-di­methyl­hydroxyl­ammonium cation forms two hydrogen bonds with the axial bromide anions of the inorganic layers as acceptors, and leads to the cohesion of the crystal structure. According to Hirshfeld surface analysis, the highest contributions to the crystal packing are from H⋯H (46.2%), Br⋯H (38.5%), and H⋯O (14.8%) contacts.

## Abstract 2

The hybrid perovskite (C_2_H_8_NO)_2_[SnBr_4_] features polymeric inorganic layers formed by corner-sharing {SnBr_6_} octa­hedra, which alternate with the organic cations.

## 1. Chemical context

Hybrid perovskites are an important class of solution-processable semiconducting materials that exhibit inter­esting electrical and optical properties. Lead halide hybrid perovskites, the most prominent members of the hybrid perovskite family, have demonstrated high power conversion efficiency in solar cells and high photoluminescence quantum yields (Sun *et al.*, 2017 ▸; Shamsi *et al.*, 2019 ▸). Despite the promising potential of lead halide hybrid perovskites, the high toxicity of lead limits their commercialization. Lead can be easily absorbed into the bloodstream and cause damage to the cardiovascular system, kidneys, reproductive system, DNA, and the central nervous system, leading to brain disorders and, ultimately, death (Jadhav *et al.*, 2007 ▸). One approach to overcoming lead toxicity is to replace lead with less toxic elements like tin or germanium, which have ionic radii similar to that of lead (Hao *et al.*, 2014 ▸).

In the past decade, tin halide hybrid perovskites have gained significant attention for solar energy applications due to their low toxicity and tunable band gap (Milot *et al.*, 2018 ▸; Li *et al.*, 2023 ▸). However, a major challenge with tin halide hybrid perovskites is the ease with which Sn^II^ oxidizes to Sn^IV^ (Byranvand *et al.*, 2022 ▸). One method to stabilize Sn^II^ is by incorporating it into the crystal structure of layered hybrid perovskites (Byranvand *et al.*, 2022 ▸). Such layered hybrid perovskites, commonly denoted as ‘two-dimensional’ hybrid perovskites, are stoichiometric compounds composed of alternating inorganic metal halide layers and organic cationic layers. This class of materials offers exceptional flexibility in terms of composition, structure, and dimensionality (Straus & Kagan, 2018 ▸). It has been shown that the hydro­phobic nature of the bulky organic cations that form the organic layers in these hybrid perovskites prevents moisture from inter­acting with the ionic inorganic layers composed of metal halide octa­hedra (Azmi *et al.*, 2024 ▸).

In this context, the search for new tin halide hybrid perovskites with a broad range of optoelectronic properties is crucial for the development of more efficient solar cells, optoelectronic, and spintronic devices. One approach to modifying the crystal structure and properties of layered hybrid perovskites is to use different templating organic cations. Hydroxyl­amines, which have been relatively underexplored, represent a promising class towards such cations. Only a few studies have reported on the synthesis of hydroxyl­amine-based metal halides (D’Annibale *et al.*, 2019 ▸; Froschauer *et al.*, 2013 ▸; Zhang *et al.*, 2018 ▸; Schottenberger *et al.*, 2015 ▸; Saal *et al.*, 2017 ▸; Ben Hmida *et al.*, 2019 ▸; Yuan *et al.*, 2019 ▸; Ban *et al.*, 1999 ▸).

In the current study, we synthesized a new layered hybrid perovskite using the reaction between *N*,*O*-di­methyl­hydroxyl­amine and tin(II) hydroxide in concentrated hydro­bromic acid. Detailed structural and Hirshfeld surface analyses of the resulting compound, (C_2_H_8_NO^+^)_2_[SnBr_4_]^−^, (**1**), were performed.
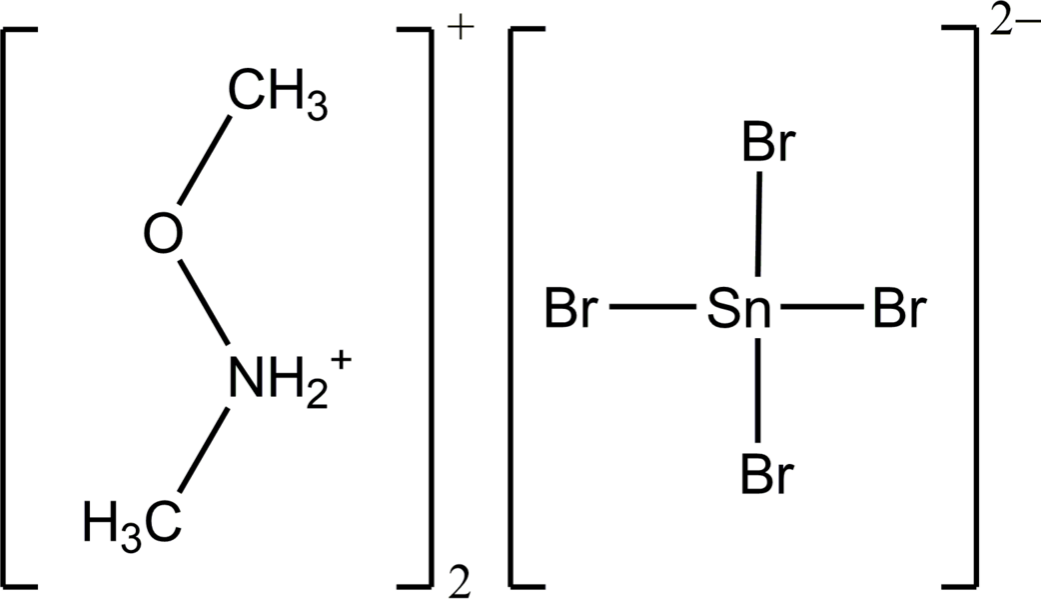


## 2. Structural commentary

**Figure 1:**
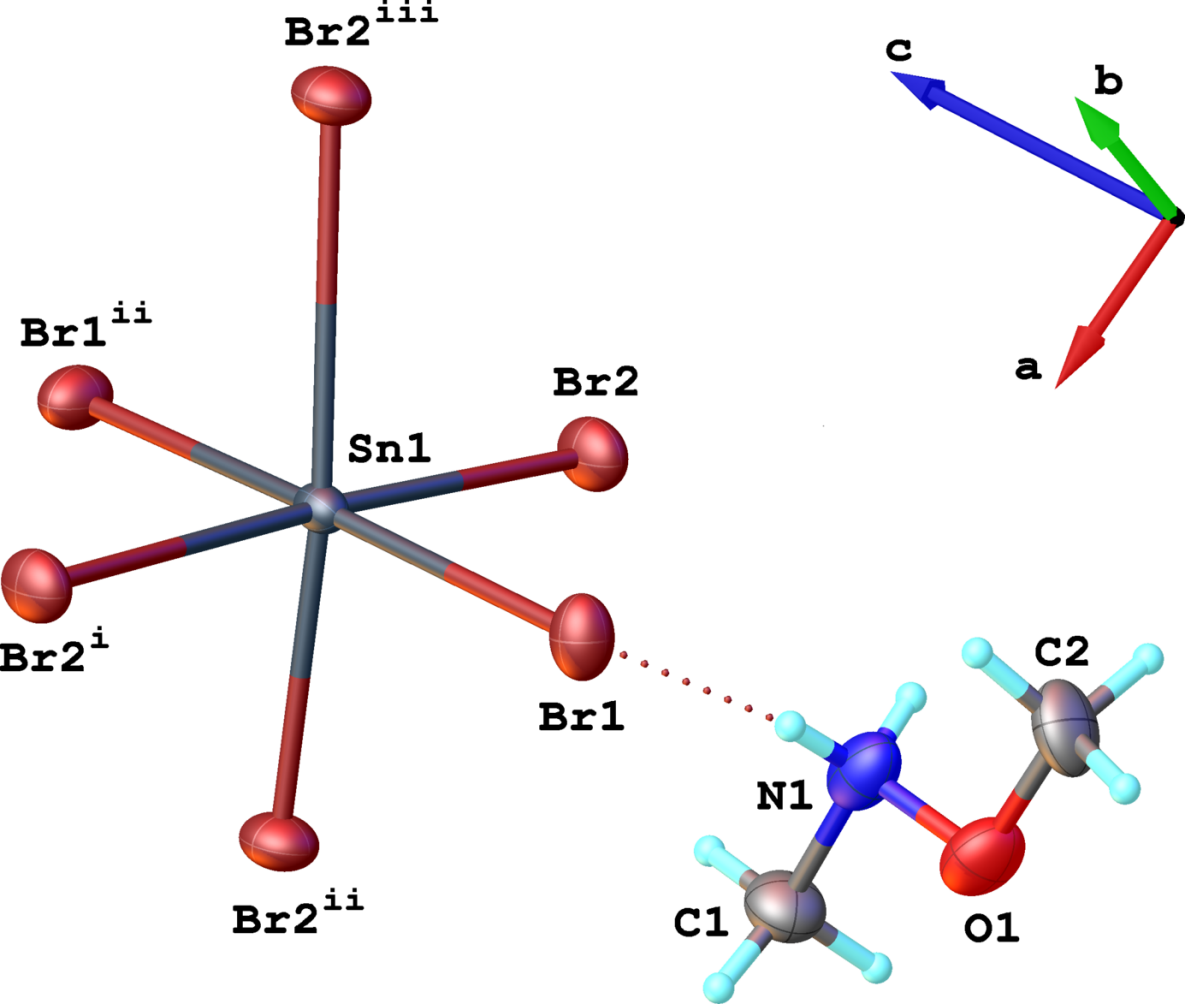
Representation of the building units in the crystal structure of compound (**1**), showing the atom-labelling scheme. Displacement ellipsoids are drawn at the 50% probability level. H atoms are shown as small spheres of arbitrary radius. [Symmetry codes: (i) 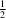 + *x*, 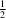 + *y*, *z*; (ii) 1 − *x*, *y*, 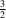 − *z*; (iii) 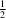 − *x*, 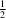 + *y*, 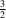 − *z*.]

Compound (**1**) has a layered crystal structure. The asymmetric unit includes one Sn^II^ atom (located on a twofold rotation axis), two Br atoms and one *N*,*O*-di­methyl­hydroxyl­ammonium cation (Fig. 1 ▸). The Sn^II^ atom is coordinated by six bromido ligands, forming a distorted {SnBr_6_} octa­hedron. The Sn—Br distances within the octa­hedron range from 2.7141 (8) Å to 3.2399 (9) Å (Table 1 ▸). Inter­estingly, a special type of octa­hedral distortion is realized, with two short equatorial Sn—Br2 bonds [2.7143 (8) Å], two long equatorial Sn—Br2 bonds [3.2395 (8) Å] and two middle-length axial Sn—Br1 bonds [2.9790 (8) Å]. The *cis*-Br—Sn—Br bond angles are in the range 84.82 (2)–98.52 (2)°, and the *trans*-Br—Sn—Br bond angles are 172.29 (3)° for equatorial Br^−^ ligands and 175.25 (4)° for axial Br^−^ ligands (Table 1 ▸), which indicates the distortion from ideal values of 90° and 180°, respectively. Qu­anti­tative octa­hedral distortion parameters can be calculated by formula Δ*d* = (1/6)Σ^6^_*i*=1_ (*d_i_*–*d*)^2^/*d*^2^ (1) and Σ = Σ^12^_*i*=1_ |90–α_*i*_| (2), where *d_i_* is the individual bond length and *d* is average bond length and α_*i*_ are twelve individual *cis*-angles in the coordination octa­hedron. The value of Δ*d* is 6.41·10^–3^, which is typical for layered tin halide hybrid perovskites (Sirenko *et al.*, 2024 ▸), and the Σ value is 39.21°.

**Figure 2:**
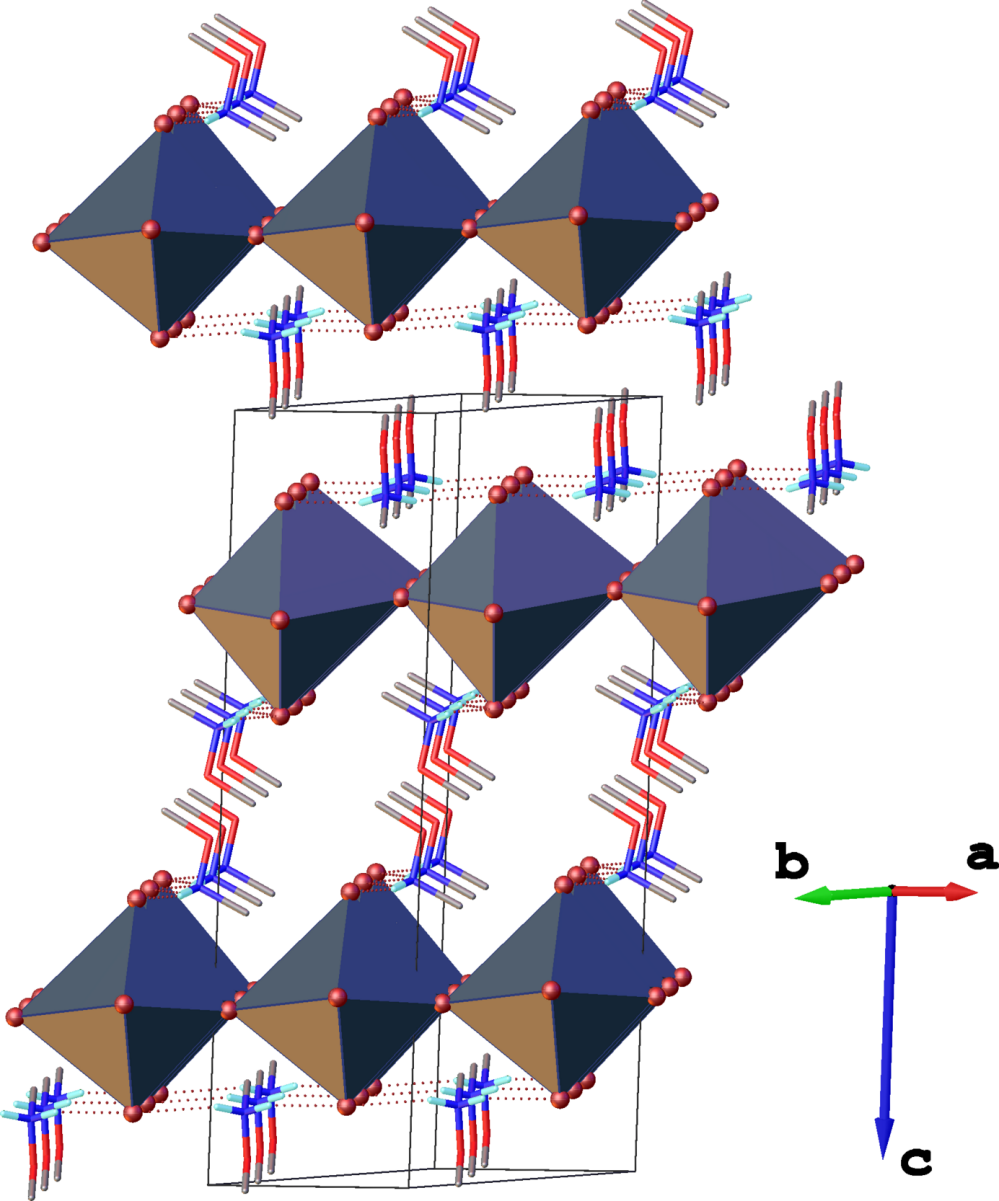
Projection of the crystal structure of (**1**) approximately along [110], showing its layered crystal structure.

The polymeric inorganic layers of (**1**) propagate parallel to the *ab* plane and are formed by sharing all equatorial corners of the {SnBr_6_} octa­hedra. These inorganic [SnBr_4/2_Br_2/1_]_∞_^2–^ layers (where Br_4/2_ denotes four equatorial atoms bonded in a corner-sharing manner and Br_2/1_ denotes two axial halogen atoms bonded only to one Sn^II^ atom) are separated by organic Me_2_HA^+^ cations (Fig. 2 ▸); the stacking direction of the layers is along the *c*-axis direction. The organic cations are aligned parallel to the *c* axis and organized in double layers (Fig. 2 ▸).

## 3. Supra­molecular features

**Figure 3:**
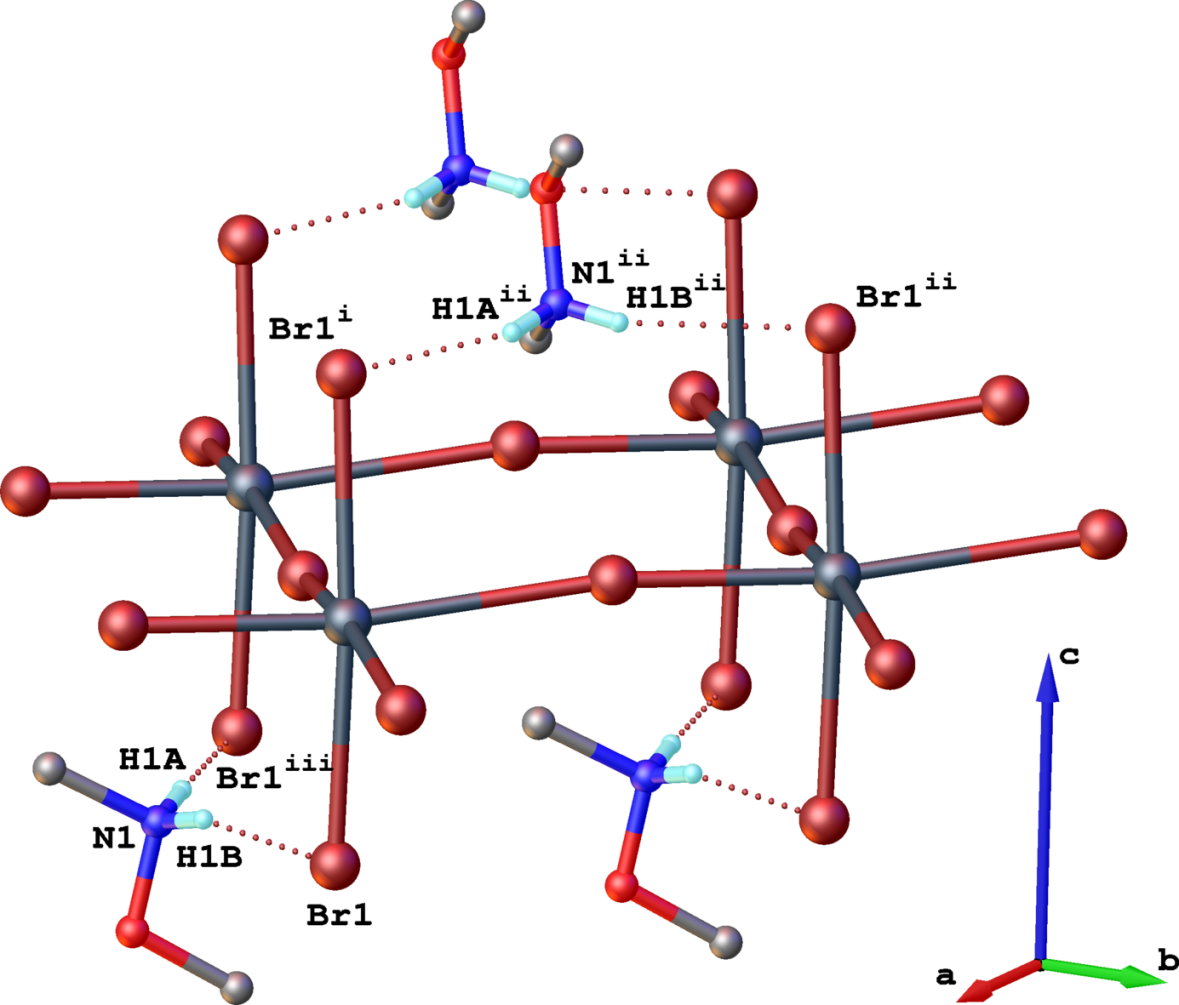
Side view of a fragment of the crystal structure of (**1**), showing the orientation of organic cations and the hydrogen-bonding scheme (dotted lines). [Symmetry codes: (i) 1 − *x*, *y*, 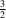 − *z*; (ii) 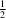 − *x*, 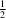 + *y*, 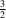 − *z*; (iii) −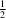 + *x*, −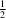 + *y*, *z*.]

The *N*,*O*-di­methyl­hydroxyl­ammonium cations inter­act with the inorganic layers through the protonated secondary amino group, forming N—H⋯Br hydrogen bonds exclusively with the axially positioned Br^−^ anions (Fig. 3 ▸, Table 2 ▸). Additionally, in the crystal structure of (**1**), weak C—H⋯Br contacts are present. As was previously shown, C—H⋯Br hydrogen bonding can be assumed when the difference (*r* C⋯B) – (*r_v_*_dw_ B + *r* C—H) < 1.00 Å, where *r* C—H is the average C—H bond length, and *r*_vdw_B is the van der Waals radius of the hydrogen-bond acceptor (Harmon *et al.*, 1992 ▸). For weak contacts C1—H1*D*⋯Br2, C2—H2*B*⋯Br1 and C2—H2*C*⋯Br1^iii^, this difference is less than 1.00 Å (0.868, 0.908 and 0.893 Å, respectively), which may indicate the presence of such weak hydrogen bonds (Table 2 ▸). While the angle for C1—H1*D*⋯Br2 (123.3°) is less characteristic of hydrogen bonding, the angles for C2—H2*B*⋯Br1 and C2—H2*C*⋯Br1^iii^ deviate less from the ideal value of 180° (Table 2 ▸), further supporting the possible presence of weak hydrogen bonding (Harmon *et al.*, 1992 ▸).

**Figure 4:**
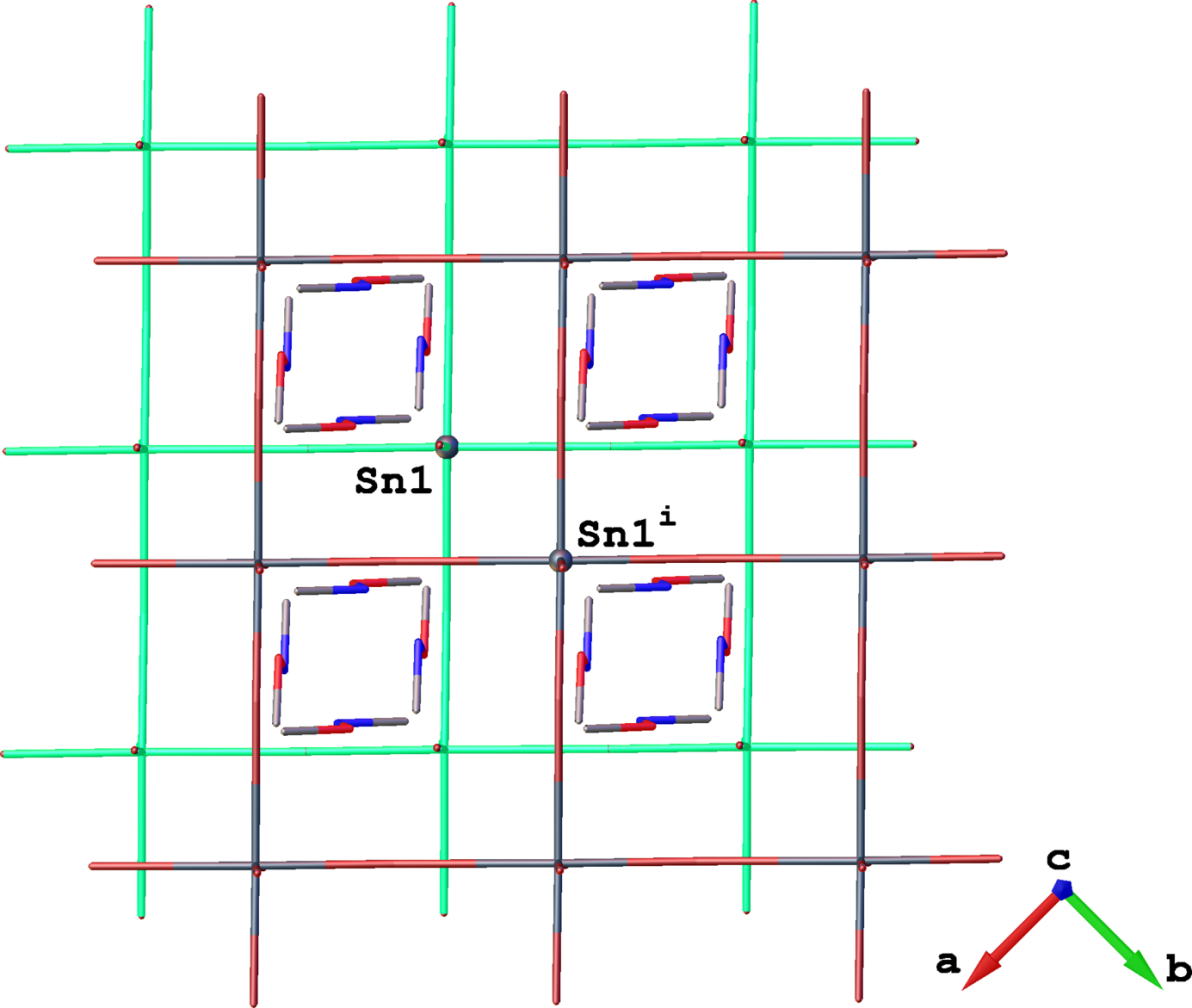
View along [001] of a fragment of inorganic layers in the crystal structure of (**1**). The upper inorganic layer is offset along the *b* axis relative to the adjacent inorganic layer (highlighted in green). In this projection, the distance between the Sn^II^ atom of the first inorganic layer is offset by 3.148 Å along the *b* axis relative to the adjacent layer. [Symmetry code: (i) *x*, 2 − *y*, 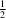 + *z*.]

It is worth noting that the *N*,*O*-di­methyl­hydroxyl­ammonium cations are oriented perpendicularly to each other on opposite sides of the inorganic layer (Fig. 3 ▸). Such an orientation of the organic cations leads to a less distorted inorganic layer compared to the case where these cations were aligned parallel to each other on both sides of the inorganic layer. This orientation of the organic cations results from the neighbouring inorganic layers positioning themselves in a manner that minimizes the free space between the Me_2_HA^+^ cations within the organic layers. The two neighbouring inorganic layers, separated by the double layers of organic cations, are shifted along the *b* axis; the Sn^II^ atom in one inorganic layer is offset by 3.148 Å along the *b* axis relative to the Sn^II^ atom located in the adjacent inorganic layer (Fig. 4 ▸).

## 4. Hirshfeld surface analysis

**Figure 5:**
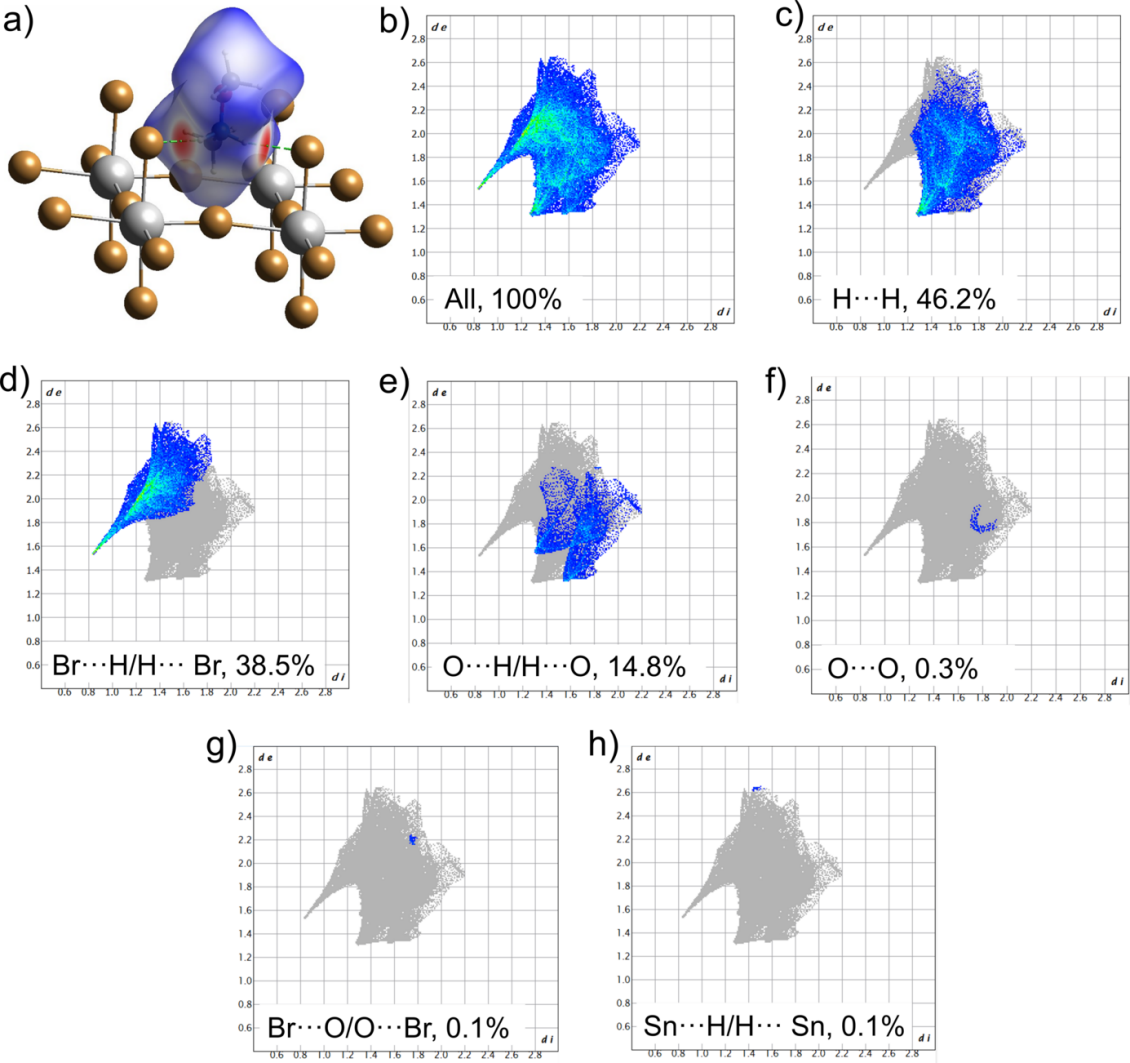
(*a*) Hirshfeld surface representation with the function *d*_norm_ plotted onto the surface for the different inter­actions; two-dimensional fingerprint plots from a Hirshfeld surface analysis of (**1**) showing: (*b*) all contacts; (*c*) H⋯H (46.2%); (*d*) Br⋯H/H⋯Br (38.5%); (*e*) O⋯H/H⋯O (14.8%); (*f*) O⋯O (0.3%); (*g*) Br⋯O/O⋯Br (0.1%) and (*h*) Sn⋯H/H⋯Sn (0.1%).

A Hirshfeld surface analysis was performed and the associated two-dimensional fingerprint plots were generated using *CrystalExplorer* (Spackman *et al.*, 2021 ▸), with standard resolution of the three-dimensional *d*_norm_ surfaces, revealing two prominent red spots and several white regions (Fig. 5 ▸). Visualizations were performed using a red–white–blue colour scheme, where red regions highlight shorter contacts, white regions indicate contacts around the van der Waals (vdW) separation, and blue areas depict longer contacts. The red spots on the Hirshfeld surface are attributed to the rather strong inter­molecular N—H⋯Br hydrogen bonds, while the white regions mainly correspond to H⋯H and O⋯H/H⋯O contacts.

The overall two-dimensional fingerprint plot (Fig. 5 ▸*b*), along with plots showing H⋯H, Br⋯H/H⋯Br, O⋯H/H⋯O, O⋯O, Br⋯O/O⋯Br, and Sn⋯H/H⋯Sn contacts and their relative contributions to the Hirshfeld surface are illustrated in Fig. 5 ▸*c–j*. The most abundant inter­action is H⋯H, which contributes 46.2% to the crystal structure of the title compound (Fig. 5 ▸*c*), with a characteristic tip at *d*_e_ = *d*_i_ = 1.3 Å. The Br⋯H/H⋯Br and O⋯H/H⋯O inter­actions contribute 38.5% and 14.8%, respectively, to the crystal structure (Fig. 5 ▸*d–e*).

The Br⋯H/H⋯Br contacts form a distinctive spike on the corresponding two-dimensional plot at (*d*_i_, *d*_e_) = (0.84, 1.53 Å), corresponding to the closest N—H⋯Br contact near 2.37 Å, indicating relevant inter­molecular inter­actions. Similarly, the O⋯H/H⋯O contacts appear as a pair of spikes at (*d*_i_, *d*_e_) = (1.55, 1.32 Å), corresponding to the closest C—H⋯O contact near 2.87 Å in the crystal structure. The contributions of the remaining O⋯O, Br⋯O/O⋯Br, and Sn⋯H/H⋯Sn inter­actions are smaller than 1.0%. The contributions of Br⋯H/H⋯Br and O⋯H/H⋯O contacts highlight the significant role of hydrogen bonding and van der Waals inter­actions in the crystal packing of (**1**).

## 5. Database survey

A search of the Cambridge Structure Database (CSD version 5.44, last update June 2023; Groom *et al.*, 2016 ▸) revealed 73 structures containing {SnBr_6_} octa­hedra and cations of organic amines. Selected examples include EKIWUS (Lorena *et al.*, 2014 ▸) and MINNOQ (Fu *et al.*, 2023 ▸), which are both layered hybrid perovskites. EKIWUS is a tin bromide compound with inorganic layers built from corner-sharing {SnBr_6_} octa­hedra, while the organic cations are organized in double layers separating the inorganic layers, similar to the title compound. By contrast, MINNOQ is a mixed lead-tin bromide hybrid perovskite featuring inorganic layers composed of corner-sharing {SnBr_6_} and {PbBr_6_} octa­hedra. Unlike EKIWUS, the organic layers in MINNOQ are organized in single layers, resulting in shorter distances between consecutive inorganic layers.

## 6. Synthesis and crystallization

SnCl_2_·2H_2_O (0.1 g, 0.44 mmol) was dissolved in 0.7 ml of distilled water with the addition of a few drops of hydro­chloric acid (HCl) to prevent hydrolysis, and the mixture was stirred until complete dissolution. Then 140 µL of NH_3_·H_2_O (35% *w*/*w*) was added to this solution, which resulted in the colourless precipitate of SnO·*x*H_2_O. The solution obtained was centrifuged at 12000 RPM for 2 min to separate SnO·*x*H_2_O from the mother liquor. The resulting solid was washed first with water and then with methanol. After each washing step, the solution was centrifuged again at 12000 RPM for 2 min. Subsequently, the obtained SnO·*x*H_2_O was allowed to dry at 303 K for 5 min before it was dissolved in a mixture of 0.5 ml concentrated HBr acid (48%_wt_) and 50 µL H_3_PO_2_ under heating and continuous stirring. Then, *N*,*O*-di­methyl­hydroxyl­amine hydro­chloride (0.89 mmol) was added to the solution. The reagents were stirred until the solution became homogenous. Light-yellow crystals were precipitated upon cooling the solution to room temperature. The crystals were separated and kept in the mother solution prior to the diffraction measurements.

## 7. Refinement

Crystal data, data collection and structure refinement details are summarized in Table 3 ▸. Methyl H atoms were positioned geometrically and refined as a rotating group, with C—H = 0.96 Å and *U*_iso_(H) = 1.5*U*_eq_(C). The H atoms of the N–H groups were positioned geometrically and refined as riding atoms with N—H = 0.80 Å and *U*_iso_(H) = 1.2*U*_eq_(N).

## Supplementary Material

Crystal structure: contains datablock(s) I. DOI: 10.1107/S2056989024012027/wm5741sup1.cif

Structure factors: contains datablock(s) I. DOI: 10.1107/S2056989024012027/wm5741Isup2.hkl

CCDC reference: 2409435

Additional supporting information:  crystallographic information; 3D view; checkCIF report

## Figures and Tables

**Table 1:** Selected geometric parameters (Å, °)

Sn1—Br1	2.9790 (8)	Sn1—Br2^i^	3.2395 (8)
Sn1—Br2	2.7143 (8)		
			
Br1—Sn1—Br1^ii^	175.25 (4)	Br2^ii^—Sn1—Br2^i^	88.972 (6)
Sn1—Br2^i^—Sn1^i^	172.29 (3)	Br1—Sn1—Br2^i^	84.82 (2)
Br2—Sn1—Br1^ii^	89.19 (3)	Br2—Sn1—Br2^ii^	91.66 (4)
Br2—Sn1—Br1	87.50 (2)	Br1^ii^—Sn1—Br2^i^	98.52 (2)

Symmetry codes: (i) 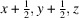; (ii) 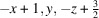.

**Table 2:** Hydrogen-bond geometry (Å, °)

*D*—H⋯*A*	*D*—H	H⋯*A*	*D*⋯*A*	*D*—H⋯*A*
N1—H1*A*⋯Br1^iii^	0.80	2.58	3.358 (6)	165
N1—H1*B*⋯Br1	0.80	2.58	3.358 (6)	165
C1—H1*D*⋯Br2	0.96	3.16	3.778 (9)	123
C2—H2*B*⋯Br1	0.96	3.04	3.818 (8)	139
C2—H2*C*⋯Br1^iii^	0.96	3.09	3.803 (9)	132

Symmetry code: (iii) 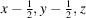.

**Table 3:** Experimental details

Crystal data	
Chemical formula	(C_2_H_8_NO)_2_[SnBr_4_]
*M*_r_	562.52
Crystal system, space group	Monoclinic, *C*2/*c*
Temperature (K)	293
*a*, *b*, *c* (Å)	8.4947 (4), 8.3064 (5), 21.6425 (14)
β (°)	96.150 (5)
*V* (Å^3^)	1518.31 (15)
*Z*	4
Radiation type	Mo *K*α
μ (mm^−1^)	12.19
Crystal size (mm)	0.20 × 0.11 × 0.09

Data collection	
Diffractometer	Xcalibur, Eos
Absorption correction	Analytical [*CrysAlis PRO* (Rigaku OD, 2023 ▸) based on expressions derived by Clark & Reid, 1995 ▸]
*T*_min_, *T*_max_	0.227, 0.438
No. of measured, independent and observed [*I* > 2σ(*I*)] reflections	6678, 1832, 1320
*R*_int_	0.047
(sin θ/λ)_max_ (Å^−1^)	0.687

Refinement	
*R*[*F*^2^ > 2σ(*F*^2^)], *wR*(*F*^2^), *S*	0.046, 0.091, 1.06
No. of reflections	1832
No. of parameters	64
H-atom treatment	H atoms treated by a mixture of independent and constrained refinement
Δρ_max_, Δρ_min_ (e Å^−3^)	0.81, −0.91

Computer programs: *CrysAlis PRO* (Rigaku OD, 2023 ▸), *SHELXT* (Sheldrick, 2015*a* ▸), *SHELXL* (Sheldrick, 2015*b* ▸), *OLEX2* (Dolomanov *et al.*, 2009 ▸) and *publCIF* (Westrip, 2010 ▸).

## supplementary crystallographic information

### catena-Poly[bis(N,O-dimethylhydroxylammonium) [di-µ-bromido-dibromidostannate(II)]] Crystal data

**Table:**

(C_2_H_8_NO)_2_[SnBr_4_]	*F*(000) = 1040
*M**_r_* = 562.52	*D*_x_ = 2.461 Mg m^−^^3^
Monoclinic, *C*2/*c*	Mo *K*α radiation, λ = 0.71073 Å
*a* = 8.4947 (4) Å	Cell parameters from 1867 reflections
*b* = 8.3064 (5) Å	θ = 3.5–26.2°
*c* = 21.6425 (14) Å	µ = 12.19 mm^−^^1^
β = 96.150 (5)°	*T* = 293 K
*V* = 1518.31 (15) Å^3^	Prism, clear light colourless
*Z* = 4	0.20 × 0.11 × 0.09 mm

### catena-Poly[bis(N,O-dimethylhydroxylammonium) [di-µ-bromido-dibromidostannate(II)]] Data collection

**Table:**

Xcalibur, Eos diffractometer	1832 independent reflections
Radiation source: fine-focus sealed X-ray tube, Enhance (Mo) X-ray Source	1320 reflections with *I* > 2σ(*I*)
Graphite monochromator	*R*_int_ = 0.047
Detector resolution: 16.1593 pixels mm^-1^	θ_max_ = 29.2°, θ_min_ = 1.9°
ω scans	*h* = −10→10
Absorption correction: analytical [CrysAlisPro (Rigaku OD, 2023) based on expressions derived by Clark & Reid, 1995]	*k* = −11→10
*T*_min_ = 0.227, *T*_max_ = 0.438	*l* = −28→27
6678 measured reflections	

### catena-Poly[bis(N,O-dimethylhydroxylammonium) [di-µ-bromido-dibromidostannate(II)]] Refinement

**Table:**

Refinement on *F*^2^	Hydrogen site location: inferred from neighbouring sites
Least-squares matrix: full	H atoms treated by a mixture of independent and constrained refinement
*R*[*F*^2^ > 2σ(*F*^2^)] = 0.046	*w* = 1/[σ^2^(*F*_o_^2^) + (0.0255*P*)^2^ + 5.9957*P*] where *P* = (*F*_o_^2^ + 2*F*_c_^2^)/3
*wR*(*F*^2^) = 0.091	(Δ/σ)_max_ = 0.001
*S* = 1.06	Δρ_max_ = 0.81 e Å^−^^3^
1832 reflections	Δρ_min_ = −0.91 e Å^−^^3^
64 parameters	Extinction correction: SHELXL-2018/3 (Sheldrick 2015b), Fc^*^=kFc[1+0.001xFc^2^λ^3^/sin(2θ)]^-1/4^
0 restraints	Extinction coefficient: 0.00064 (10)
Primary atom site location: dual	

### catena-Poly[bis(N,O-dimethylhydroxylammonium) [di-µ-bromido-dibromidostannate(II)]] Special details

**Table:**

Geometry. All esds (except the esd in the dihedral angle between two l.s. planes) are estimated using the full covariance matrix. The cell esds are taken into account individually in the estimation of esds in distances, angles and torsion angles; correlations between esds in cell parameters are only used when they are defined by crystal symmetry. An approximate (isotropic) treatment of cell esds is used for estimating esds involving l.s. planes.

### catena-Poly[bis(N,O-dimethylhydroxylammonium) [di-µ-bromido-dibromidostannate(II)]] Fractional atomic coordinates and isotropic or equivalent isotropic displacement parameters (Å^2^)

**Table:**

	*x*	*y*	*z*	*U*_iso_*/*U*_eq_	
Sn1	0.500000	0.81057 (7)	0.750000	0.0296 (2)	
Br1	0.49095 (9)	0.79570 (10)	0.61214 (3)	0.0527 (3)	
Br2	0.26948 (8)	0.58288 (9)	0.74077 (4)	0.0525 (3)	
O1	0.3786 (7)	0.3817 (7)	0.5434 (3)	0.0657 (16)	
N1	0.3672 (7)	0.4115 (8)	0.6048 (3)	0.058 (2)	
H1A	0.280 (6)	0.3942 (13)	0.6130 (6)	0.069*	
H1B	0.3886 (15)	0.503 (6)	0.6129 (6)	0.069*	
C2	0.2663 (9)	0.4871 (10)	0.5057 (4)	0.065 (2)	
H2A	0.291904	0.488360	0.463565	0.097*	
H2B	0.273286	0.594386	0.522265	0.097*	
H2C	0.160523	0.447150	0.506703	0.097*	
C1	0.4823 (9)	0.3014 (10)	0.6406 (4)	0.060 (2)	
H1C	0.461883	0.192512	0.627173	0.090*	
H1D	0.470815	0.310042	0.684117	0.090*	
H1E	0.588140	0.330831	0.633490	0.090*	

### catena-Poly[bis(N,O-dimethylhydroxylammonium) [di-µ-bromido-dibromidostannate(II)]] Atomic displacement parameters (Å^2^)

**Table:**

	*U*^11^	*U*^22^	*U*^33^	*U*^12^	*U*^13^	*U*^23^
Sn1	0.0243 (3)	0.0241 (3)	0.0401 (4)	0.000	0.0021 (3)	0.000
Br1	0.0573 (5)	0.0561 (6)	0.0440 (5)	−0.0174 (4)	0.0018 (4)	0.0028 (4)
Br2	0.0414 (4)	0.0440 (5)	0.0722 (6)	−0.0117 (3)	0.0061 (4)	−0.0013 (4)
O1	0.075 (4)	0.069 (4)	0.058 (4)	−0.003 (3)	0.026 (3)	−0.012 (3)
N1	0.062 (4)	0.052 (4)	0.063 (5)	−0.016 (3)	0.022 (4)	−0.014 (4)
C2	0.067 (5)	0.071 (6)	0.051 (5)	−0.008 (5)	−0.021 (4)	0.013 (5)
C1	0.056 (5)	0.055 (5)	0.067 (6)	0.014 (4)	−0.003 (4)	0.007 (5)

### catena-Poly[bis(N,O-dimethylhydroxylammonium) [di-µ-bromido-dibromidostannate(II)]] Geometric parameters (Å, º)

**Table:**

Sn1—Br1^i^	2.9791 (8)	N1—H1B	0.80 (5)
Sn1—Br1	2.9790 (8)	N1—C1	1.493 (9)
Sn1—Br2^i^	2.7144 (8)	C2—H2A	0.9600
Sn1—Br2	2.7143 (8)	C2—H2B	0.9600
Sn1—Br2^ii^	3.2395 (8)	C2—H2C	0.9600
O1—N1	1.365 (8)	C1—H1C	0.9600
O1—C2	1.476 (9)	C1—H1D	0.9600
N1—H1A	0.80 (5)	C1—H1E	0.9600
			
Br1—Sn1—Br1^i^	175.25 (4)	C1—N1—H1A	110.4
Sn1—Br2^ii^—Sn1^ii^	172.29 (3)	C1—N1—H1B	110.4
Br2—Sn1—Br1^i^	89.19 (3)	O1—C2—H2A	109.5
Br2—Sn1—Br1	87.50 (2)	O1—C2—H2B	109.5
Br2^i^—Sn1—Br2^ii^	88.972 (6)	O1—C2—H2C	109.5
Br1—Sn1—Br2^ii^	84.82 (2)	H2A—C2—H2B	109.5
Br2^i^—Sn1—Br1	89.19 (3)	H2A—C2—H2C	109.5
Br2^i^—Sn1—Br1^i^	87.50 (2)	H2B—C2—H2C	109.5
Br2—Sn1—Br2^i^	91.66 (4)	N1—C1—H1C	109.5
Br1^i^—Sn1—Br2^ii^	98.52 (2)	N1—C1—H1D	109.5
N1—O1—C2	108.8 (6)	N1—C1—H1E	109.5
O1—N1—H1A	110.4	H1C—C1—H1D	109.5
O1—N1—H1B	110.4	H1C—C1—H1E	109.5
O1—N1—C1	106.4 (6)	H1D—C1—H1E	109.5
H1A—N1—H1B	108.6		
			
C2—O1—N1—C1	179.7 (6)		

Symmetry codes: (i) −*x*+1, *y*, −*z*+3/2; (ii) *x*+1/2, *y*+1/2, *z*.

### catena-Poly[bis(N,O-dimethylhydroxylammonium) [di-µ-bromido-dibromidostannate(II)]] Hydrogen-bond geometry (Å, º)

**Table:**

*D*—H···*A*	*D*—H	H···*A*	*D*···*A*	*D*—H···*A*
N1—H1*A*···Br1^iii^	0.80	2.58	3.358 (6)	165
N1—H1*B*···Br1	0.80	2.58	3.358 (6)	165
C1—H1*D*···Br2	0.96	3.16	3.778 (9)	123
C2—H2*B*···Br1	0.96	3.04	3.818 (8)	139
C2—H2*C*···Br1^iii^	0.96	3.09	3.803 (9)	132

Symmetry code: (iii) *x*−1/2, *y*−1/2, *z*.
